# Guardians of the Herd: Molecular Surveillance of Tick Vectors Uncovers Theileriosis Perils in Large Ruminants

**DOI:** 10.3390/microorganisms11112684

**Published:** 2023-11-02

**Authors:** Muhammad Sohail Sajid, Asif Iqbal, Hafiz Muhammad Rizwan, Asma Kausar, Urfa Bin Tahir, Muhammad Younus, Mahvish Maqbool, Rao Muhammad Siddique, Dalia Fouad, Farid Shokry Ataya

**Affiliations:** 1 Department of Parasitology, Faculty of Veterinary Science, University of Agriculture, Faisalabad 38000, Pakistan; dr.urfa@uaf.edu.pk (U.B.T.); mmabdullah017@gmail.com (M.M.); 2 Department of Parasitology, Riphah College of Veterinary Sciences, Lahore 54000, Pakistan; aiqbal.rcvets@riphah.edu.pk (A.I.); drraosiddique@gmail.com (R.M.S.); 3 Section of Parasitology, Department of Pathobiology, Khan Bahadur Chaudhary Mushtaq Ahmad College of Veterinary and Animal Sciences, Narowal, Sub Campus UVAS, Lahore 54000, Pakistan; 4 Livestock and Dairy Development (L&DD), Veterinary Research Institute, Lahore 54080, Pakistan; asmakausar20@gmail.com; 5 Department of Aquatic Animal Medicine, College of Fisheries, Huazhong Agricultural University, Wuhan 430070, China; 6 Section of Pathology, Department of Pathobiology, Khan Bahadur Chaudhary Mushtaq Ahmad College of Veterinary and Animal Sciences, Narowal, Sub Campus UVAS, Lahore 54000, Pakistan; younusrana@uvas.edu.pk; 7 Department of Zoology, College of Science, King Saud University, P.O. Box 22452, Riyadh 11459, Saudi Arabia; dibrahim@ksu.edu.sa; 8 Department of Biochemistry, College of Science, King Saud University, P.O. Box 2455, Riyadh 11451, Saudi Arabia; fataya@ksu.edu.sa

**Keywords:** *Hyalomma anatolicum*, *Theileria annulate*, PCR, tick vector, blood, ruminants

## Abstract

*Theileria* sp. (Piroplasmida: Theileriidae) is one of the most widely known infections transmitted by hard ticks (Acari: Ixodidae) and has been linked to significant economic losses across the globe. The study’s main emphasis was theileriosis, a disease that is common in Pakistan and has an incidence ranging from 0.6% to 33%. Through DNA screening of the vector ticks and host blood, this study sought to determine the risk of tick-borne theileriosis in populations of buffalos (*Bubalus bubalis*) and cattle (*Bos indicus*) in Toba Tek Singh district of Punjab, Pakistan. Identified tick species include *Hyalomma anatolicum* (35.4%), *Rhipicephalus (Boophilus) microplus* (30.2%), and *R. sanguineus* (25%). Tick specimens were collected from animals and their respective microenvironments. PCR assays targeting *Theileria annulata* were used to investigate the infection in the DNA extracted from the collected blood samples from large ruminants and salivary glands (SGs) of the *Hyalomma* ticks. The 18S rRNA of *T. annulata* was amplified using specific primers. Positive *T. annulata* amplicons were sequenced and verified using BLAST analysis. Overall, 50% of SGs contained *T. annulate* DNA. Female ticks, and those collected from cattle and from riverine environments had significantly higher (*p* < 0.05) rates of *Theileria* infection in their acini. Overall prevalence of *Theileria* infection was 35.9% in blood collected from large ruminants. Cattle had a substantially greater frequency of bovine theileriosis (43.2%) than buffalos (28.7%). Age and sex of large ruminants were significantly positively associated (*p* < 0.05) with *Theileria* infection. Furthermore, compared to non-riverine cattle (35%) and buffalo (19.5%), riverine cattle (52.2%) and buffalo (36.2%) showed a considerably higher prevalence. The results of this study, which is the first in Pakistan to examine the blood of large ruminants and vectorial function of Ixodid ticks in the transmission of *T. annulata* along with associated risk factors, offer an important insight for risk assessment of *Theileria* infection in livestock using vectorial infectivity.

## 1. Introduction

In addition to restlessness and anemia in animals, ticks (Acari: Ixodidae) also act as carriers of viruses, bacteria, protozoa, and rickettsia, among other pathogens. Tick infestation poses serious danger to the livestock in many parts of the world (Asmaa et al., 2014 [1]). Compared to other tick species, *Hyalomma* spp. (Ixodidae) have a remarkable capacity to survive in severe and difficult environments (Hasan et al., 2012 [2]). The majority of *Hyalomma* spp., including *Hyalomma anatolicum, H. scupense*, and *H. lusitanicum*, are known to transmit tropical theileriosis due to *Theileria annulata* (Piroplasmida: Theileriidae) (Yamchi and Tavassoli, 2016 [3]). Unfortunately, many factors have led to dangerously high levels of *H. anatolicum* in Pakistan (Rehman et al., 2017 [4]). Pakistan’s livestock industry is seriously threatened by *Hyalomma* and *Rhipicephalus* tick spp. *Babesia bovis*, *B. bigemina*, and *Anaplasma marginale* are among the major tick-borne diseases in Pakistan, and they are prevalent throughout the world too (Perveen, 2011; Karim et al., 2017 [5,6]). In three temporal zones of KPK (Khyber Pakhtunkhwa), *R. annulatus* is the dominant species, followed by *R. microplus* and *Haemaphysalis aciculifer* (Farooqi et al., 2017 [7]). A critical step in risk assessment of *Theileria* species in domestic animals is through the examination of infesting tick populations. A simple way to predict the danger of theileriosis in livestock and the spread of the disease is to screen tick salivary glands (SGs) for *Theileria* sporozoites (Amiri et al., 2021; Zeb et al., 2022 [8,9]).

Theileriosis, a serious protozoal parasitic disease of ruminants, is caused by various species of the genus *Theileria* (Jabbar et al., 2015 [10]). Depending on the infecting species, the infected ruminants experience a condition known as tropical theileriosis or East Coast fever (Jabbar et al., 2015; Fry et al., 2016 [10,11]) and may display clinical signs such a high temperature, anemia, anorexia, and decreased milk output. The infections may be fatal in extreme circumstances (Islam et al., 2011 [12]). As a result of decreased productivity, higher veterinary expenses, and potential death, the financial effects on herds can be overwhelming to livestock operations. According to Brown (1997 [13]), this condition estimates significant economic losses of up to US $800 million per year in India, which are attributable to decreased production, morbidity, mortality, the use of anti-theilerial medications and tick control techniques such acaricides, and cost of other management strategies.

On a global scale, efforts have been undertaken to identify theilerial sporozoites by staining tick SGs with methyl green pyronin (MGP); however, the precise tick species vector remains unknown (De La Fuente et al., 2008 [14]). It is typically impossible to morphologically distinguish *T. annulata* from other *Theileria* spp. that are not harmful. Thus, *Theileria* sporozoites in vectors have been identified using polymerase chain reaction (PCR) (Amiri et al., 2021 [8]). The purpose of this work was to use PCR to determine the magnitude of *Theileria* infection in the SGs of questing (off-host) and infesting (on-host) tick populations collected from riverine (habitats located close to or along the banks of rivers) and non-riverine (habitats located at a distance from rivers) areas and in blood samples of large ruminants and their association with various factors in Toba Tek (T.T.) Singh district of Punjab, Pakistan.

## 2. Materials and Methods

### 2.1. Study Area and Collection of Ticks

Three tehsils make up the T.T. Singh district (30.98° N, 72.48° E): T.T. Singh, Kamalia, and Gojara. In the research area, adult and partially fed Ixodid ticks were collected from buffalos (*Bubalus bubalis*) and cattle (*Bos indicus*) that appeared to be in good health. For additional processing, ticks were brought to the Department of Parasitology at the University of Agriculture in Faisalabad, Pakistan. The study was conducted for a period of six months, from March 2022 to August 2022.

### 2.2. Collection of Blood

Blood samples were taken from both tick-infested and non-infested animals. Using a sterile syringe and ethylene-diamine tetraacetic acid (EDTA)-filled test tube, a blood sample (10 mL) was drawn aseptically from the jugular vein. Samples were transported to the laboratory and stored in a deep freezer at 4 °C for DNA extraction. Each animal’s information (tag number, breed, etc.) was recorded in a separate file. *Theileria* DNA was detected through PCR analysis of the blood, as described below.

This study was approved by the Research Ethics Committee, Faculty of Veterinary Science, University of Agriculture, Faisalabad, Pakistan, and University of Veterinary and Animal Sciences, Lahore, Pakistan. The standard guidelines for institutional animal care and use (IACU), University of Agriculture, Faisalabad, Pakistan, were followed.

### 2.3. Identification and Dissections of Ticks

The keys developed by Walker et al. (2003 [15]) were used to identify ticks. SGs were obtained by stereoscopic dissections of *H. anatolicum* ticks as described by Purnell et al. (1968 [16]). Dissections were carried out in ice-cold phosphate-buffered saline (PBS), pH 7. Individual ticks with at least 8 or more acini were analyzed for *Theileria* infection to determine the intensity of infection.

### 2.4. DNA Extraction and Amplification

Following the manufacturer’s instructions, blood from host and SGs from ticks were used for genomic DNA extraction using a DNeasy kit (Qiagen, Hilden, Germany). Quantification of extracted DNA was based on the ratio of absorbance at 260/280 nm using a nanodrop spectrophotometer (Nanodrop Technologies, Wilmington, DE, USA), then it was stored at −20 °C until use. Based on host species, tick gender, infestation status of ticks (infesting or questing), and ecological variables, pools were made. Using the oligonucleotide primer sets for *Theileria* genus, (Ta F) 5′AGT TTC TGA CCT ATC AG3′ and (Ta R) 5′TTG CCT TAA ACT TCC TTG3′ (Allsopp et al., 1993 [17]), and for *T. annulate*, (Ta F) 5′GTA ACC TTT AAA AAC GT3′ and (Ta R) 5′GTT ACG AAC ATG GGT TT3′ (d’Oliveira et al., 1995 [18]), PCR experiments amplified a segment of the 18S rRNA gene. A 25 µL reaction volume consisted of 12.5 µL PCR supermix (Catalog number: 10572014) with 2 mM MgCl_2_, 0.05 u/µL Taq DNA polymerase, 0.2 mM of each deoxynucleotide triphosphate (Catalog number: PR-C1141), 0.5 µL forward and reverse primers, 4.5 µL nuclease-free water (Catalog number: AM9932), and 7 µL extracted DNA. DNA amplification began with an initial denaturation step at 94 °C for 5 min. Subsequently, 40 amplification cycles were performed, each consisting of denaturation at 94 °C for 1 min, annealing at 58 °C for 1 min, and extension at 72 °C for 1 min. This amplification process was carried out in a thermal cycler (C1000 Thermal Cycler, Bio Rad, Hercules, CA, USA). Ten minutes at 72 °C were spent on the last extension, then it was ended by cooling at 4 °C. PCR products were electrophoresed through a 1.8% agarose gel with an ethidium bromide (1 µg/mL) indicator in a running buffer containing Tris Acetate EDTA at 90 V for 45–60 min. Molecular Imager^®^ Gel DocTM XR+ with Image LabTM software version 6.1 was used to image gels after transfer to a gel documentation system (Bio Rad, Hercules, CA, USA). Purified PCR products were sequenced for validation, then a homology search was conducted on the NCBI website.

### 2.5. Statistical Analyses

The Chi-square test (categorical variables) was used to examine differences between independent factors (host, sex, infestation status, and ecological variable) with respect to prevalence of *Theileria* infection. The software SPSS 17.0 (SPSS Inc., Chicago, IL, USA) was used to analyze the data. By dividing the number of positive samples by the total number of samples and then multiplying the result by 100, the relative prevalence of *Theileria* infection was determined.

## 3. Results

**Figure 1:**
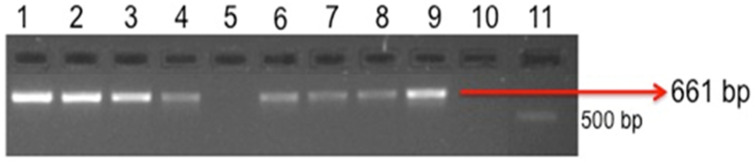
Amplification of *T. annulata* from salivary glands of ticks. Lane No. 1, 2, 3, 4, 6, 7, and 8 = positive for *T. annulata*, Lane No. 5 = negative for *T. annulata*, Lane No. 9 = positive control *T. annulata*, Lane No. 10 = negative control *T. annulata*, and Lane No. 11 = 1 kb DNA ladder.

Among the collected ticks, the highest (*p* < 0.05) prevalence was of *H. anatolicum* (35.4%), followed in order by *R. microplus* (30.2%) and *R. sanguineus* (25%) (Table 1). Due to our focused hypothesis of *Hyalomma* infectivity for *Theileria* spp., we only dissected SGs of *Hyalomma* spp. for PCR. In total, 126 of the 252 pools (50%) of *Hyalomma* spp. SGs were positive for *T. annulate* (Figure 1). After DNA sequencing of representative positive samples, 100% homology with *T. annulata* was found through BLAST analysis.

**Figure 2:**
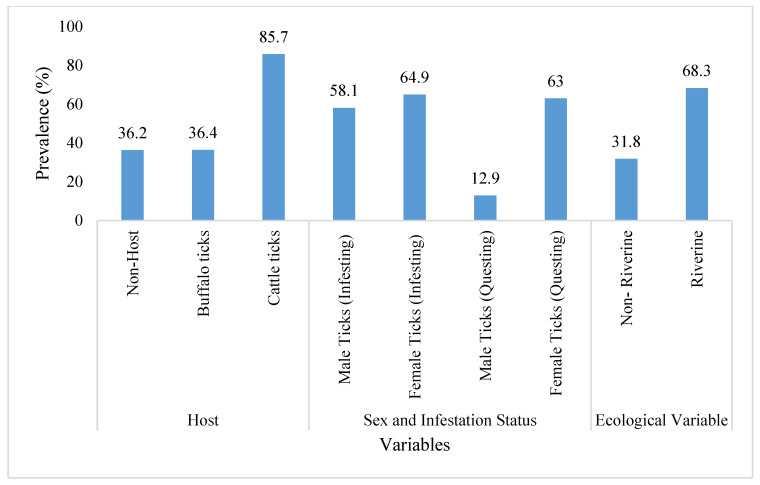
Prevalence of *Theileria* infection in tick populations in Toba Tek Singh district, Punjab, Pakistan.

The frequency distribution of *Theileria* DNA in the SGs of *H. anatolicum* ticks is shown in Table 2. Compared to ticks obtained from buffalos, ticks infesting cattle showed a higher prevalence (*p* < 0.05) of *Theileria* infection. Additionally, for both infesting and questing ticks, the infection was significantly higher (*p* < 0.05) in female tick acini than males. Similarly, infesting (on-host) ticks were more likely to be infected with *Theileria* than questing ticks (*p* < 0.05). The prevalence of *Theileria* infection was significantly higher (*p* < 0.005) in ticks collected from riverine compared to non-riverine areas (Table 2, Figure 2).

Bovine tropical theileriosis was found in 35.9% of cases. In total, 84 of the 136 animals whose ticks were screened were positive for *Theileria*. Out of these, all 84 came from animals whose blood samples also tested positive for *Theileria*, demonstrating a direct link between tick and blood infection. Cattle and buffalos were both *Theileria*-infected. Cattle (43.23%) had a significantly higher frequency of theileriosis than buffalos (28.7%). Theileriosis was most prevalent in Friesian cattle (57.9%), followed in order by Jersey (44.9%) and Sahiwal (35.3%) breeds. Theileriosis was more common in Kundi buffalo (44.9%) than Nili Ravi breed (17.5%). Bovine tropical theileriosis was more prevalent in female cattle and buffalos (prevalence rates of 47.9% and 31.6%, respectively) than in males (prevalence rates of 28.3% and 16.2%, respectively).

Additionally, compared to adult cattle (36.1%) and buffalos (23.1%), the prevalence of theileriosis was significantly higher in calves of both cattle (55.7%) and buffalos (40.3%). Furthermore, riverine cattle had a higher prevalence (52.2%) than non-riverine cattle (35%). Similarly, riverine buffalos (36.2%) had a higher prevalence than non-riverine buffalos (19.5%) (Table 3).

## 4. Discussion

A significant component of the livestock industry, which is essential to Pakistan’s agricultural economy, is large ruminants. Livestock production is at risk due to tick-borne infections such as *T. annulata*, which are indigenous to tropical and subtropical regions of the world, including Pakistan. Tropical theileriosis causes the livestock industry in underdeveloped nations significant financial losses (Rehman et al., 2019; Zeb et al., 2020 [19,20]).

Tick infestations vary across different regions of the world, including Asia (Batool et al., 2022; Zeb et al., 2022 [9,21]), Australia (Islam et al., 2011 [12]), Africa (Morrison et al., 2020 [22]), Europe (Liu et al., 2021 [23]), and the Americas (Almazán et al., 2022 [24]). In Faisalabad, Pakistan, tick species prevalent among large ruminant populations include *Dermacentor marginatus*, *R. annulatus*, *R. sanguineus*, *R. microplus*, *H. anatolicum*, and *H. aegyptium* (Khan et al., 1993 [25]). In districts Muzaffargarh and Layyah, ticks such as *H. anatolicum* and *R. sanguineus* were reported (Sajid et al., 2009 [26]), while NWFP exhibited *D. raskimensis*, *R. sharifi*, *R. microplus*, *Haemaphysalis* (*Ha.*) *montgonervi*, *Ha. cornupunctata*, *H. anatolicum*, *H. scupense*, *H. excavatum*, *H. marginatum*, *H. dromedarii*, *R. haemaphysaloides*, and *R. sanguineus* (Siddiqi et al., 1986 [27]). Sixteen tick species were reported in Sindh province, with the two most common being *R. turanicus* and *H. anatolicum* (Hussain et al., 1986 [28]). According to studies by Iqbal et al. (2013 [29]) and Rehman et al. (2017 [4]), the genus *Hyalomma* has been remarkably common in domestic livestock populations in Pakistan. The predilection of *H. anatolicum* for cattle (Islam et al., 2011 [12]) and the innate immunity arising from genetic differences between cattle and buffalo, as well as variations within cattle breeds, may be responsible for the frequent infestation of cattle by *Hyalomma* spp. of ticks.

The acini of female tick SGs were more frequently *T. annulata*-infected than those of male ticks. This is consistent with the finding that female ticks have greater frequencies of *Theileria* infection (Yamchi and Tavassoli, 2016 [3]). The different feeding habits of male and female ticks may account for variation in infection rates. Male ticks are sporadic feeders; they frequently attach to the host, feed early, and then detach to mate with females. If females are not present, male ticks may either stay connected to the host or drop off. While this is not directly related to the infection rate of females, it does imply that males may feed earlier and more erratically, which could facilitate the sporogony (Kocan et al., 1988 [30]). Males may then transmit sporozoites to the host during the first few days of attachment before mating with females. Due to early sporozoite transfer to the host, SGs of male ticks may have lower observed infection rates. To comprehend the mechanisms underlying the different infectivity rates of males and females, however, more research is required (Woods et al., 2021 [31]). The differential tick infection rates across the sexes raise interesting issues regarding the biology of these ticks. It is possible that hormonal or physiological variations between male and female ticks account for the increased infection rates seen in female tick acini. Investigating the biological differences between male and female ticks may provide vital information about the dynamics of the transmission of pathogens carried by ticks.

An interesting finding was that cattle had a considerably higher rate of *Theileria* infection than buffalos, which may indicate host susceptibility or vector preference differences for the parasite. In line with earlier studies (Kolte et al., 2017 [32]), we found that *Theileria* sporoblast prevalence was higher in ticks collected from cattle than from buffalos in the same region. It is crucial to remember that this does not suggest that the host is the cause of the higher *Theileria* infectivity in *H. anatolicum* ticks, but rather that the higher infestation rate of *H. anatolicum* on cattle compared to buffalos may be responsible. Additionally, because their nymphal stage may feed on calves showing high parasitemia, *Hyalomma* ticks that infest cattle may be more frequently infected (Kumar et al., 2020 [33]). This result confirms that crossbred cattle are more susceptible to *Theileria* spp. than buffalos, perhaps as a result of higher vector susceptibility of the former (Sajid et al., 2009 [26]). To estimate the risk posed to vertebrate hosts, it is essential to evaluate the prevalence of *Theileria* spp. in infesting and questing tick populations. To quantify the risk of tropical theileriosis in cattle and buffalos in the research area, our determination of *Theileria* spp. distribution in tick vectors is extremely helpful. It is possible that this variation results from a variety of variables, such as variations in these animal species’ tick feeding habits or host immune responses. Understanding these elements may shed light on the mechanisms underlying the host’s vulnerability to *Theileria* infections and open the door to specialized treatment plans.

*Theileria annulata*, which causes tropical theileriosis and is mostly transmitted by *H. anatolicum* ticks in Pakistan, was most prevalent in tick pools (6.7%), followed by *T. orientalis* (3.5%) and *T. ovis* (0.2%), according to Rehman et al. (2019 [20]). The most virulent of these species, *T. annulata*, has a large number of strains that are widely dispersed over the globe. In Africa and Asia, the dairy industry suffers significant financial losses from *T. annulata*, which causes a serious and potentially deadly disease in calves (Bishop et al., 2009; Haque et al., 2010 [34,35]). According to Jabbar et al. (2015 [10]), the disease is more severe in exotic and cross-bred cattle, in which the case–fatality rate can reach up to 80%, vs. 20% in native breeds.

Analysis by age revealed that young ruminants were more susceptible to *Theileria* infection than adults. Age as a risk factor yielded notable outcomes as well. These results are in line with previous studies (Qayyum et al., 2010, Farooqi et al., 2017, Zeb et al., 2020, Ullah et al., 2021 [19,36,37,38]) that revealed a similar profile of *Theileria* infection from semi-arid and arid agro-climatic zones of the country. The owner of the herd may have taken better care of adult animals, especially dairy animals, while neglecting young ones, which may be the cause of the higher incidence in young ruminants. Young animals’ immune systems are not fully developed to resist or limit infection with *T. annulata* (Kabir et al., 2011 [39]). On the other hand, older animals may have a lower prevalence of *Theileria* because of a lifetime history of immune development and recurring infections (Ilhan et al., 1998; Gharbi and Darghouth, 2014 [40,41]). Bovine tropical theileriosis, with breed- and age-related variations, was common in both cattle and buffalos. These insights are essential for the creation of focused strategies for the control of ticks and tick-borne diseases in the area.

There were also gender-related differences, with female ticks and female ruminants having greater infection rates. These results call for more research into *Theileria*-specific interactions between ticks and gender of vector and hosts. Valente et al. (2023 [42]) found a greater proportion of females with *T. annulata* infection, similar to our study. The findings of Valente et al. (2023 [42]) contradicted our findings, which showed no statistically significant correlation between *T. annulata* positivity and sex. This could be explained by the fact that we studied a significantly higher proportion of females than males. This may be the outcome of immunosuppression in female animals brought on by ongoing stress and hormonal changes during nursing and pregnancy. Additionally, this is consistent with the findings of Kamani et al. (2010 [43]) and Parveen et al. (2021 [44]), which point to a higher prevalence in females due to their prolonged retention for various functions, such as reproduction and milk production, as well as the possibility that they may not receive sufficient feed to satisfy the high nutritional demands associated with their reproductive function.

Due to the complex relationship of ecological conditions, riverine ticks have a higher prevalence of *Theileria* infection than non-riverine ticks. Both tick vectors and *Theileria* parasites are prevalent in riverine habitats. Ticks have more possibilities to feed in these places since there are more compatible hosts available, which increases their likelihood of contracting and spreading the parasite (Salih et al., 2003; Magzoub et al., 2021 [45,46]). Additionally, ticks found in riverine areas may have greater *Theileria* vector competency, increasing the incidence of disease transmission. *Theileria* infectivity in ticks within riverine habitats may also be more prevalent due to regional livestock management practices and the existence of wildlife reservoirs (Iweriebor et al., 2022 [47]). The need for focused surveillance and control strategies to handle tick-borne diseases in such areas is successfully highlighted by these complicated ecological dynamics.

## 5. Conclusions

In conclusion, ecological investigations coupled with molecular screening tools used in the current investigations offered significant information about factors that influence tick prevalence and *Theileria* infection, such as species, breed, age, and sex of host, sex and infestation status of ticks, and ecological variables, as well as new insights into the PCR-based vectorial capacity for risk assessment in the community. The results of this comprehensive study shed light on the prevalence, patterns of distribution, and host connections of the complex web of tick-borne diseases. *Hyalomma anatolicum* is the most common species of tick, highlighting the wide habitat that ticks survive in. This diversity emphasizes the necessity for sophisticated approaches that take into account the unique ecological niches occupied by various vectors in order to combat diseases carried by ticks. Our data on the abundance of *H. anatolicum* and *T. annulata* in the host blood and vector ticks in the area emphasize how crucial it is to institute specific tick and theileriosis control methods. A stunning 50% positivity rate, verified by PCR, for the presence of *T. annulata* in tick salivary glands illustrates the importance of this point. The accuracy of these results is further supported by the 100% homology revealed by BLAST analysis. This confirms that *H. anatolicum* ticks are *T. annulata* carriers and highlights the risk they pose to large ruminants in the research area.

Compared to those found on buffalos, ticks infesting cattle had a greater infection rate. On-host ticks were more likely to be infected than questing ticks, and female tick acini were noticeably more contaminated than male ones. Furthermore, compared to ticks from non-riverine locations, those collected from riverine areas had higher infection rates. Bovine tropical theileriosis was found in 35.9% of cases when it was investigated. A total of 84 out of 136 animals had positive results for both tick and blood samples, proving the causal relationship between tick and blood infection. Geographically, ticks from riverine areas had greater infectivity rates, highlighting the significance of taking environment into account when analyzing disease dynamics. This study provides important information about the prevalence and variables affecting *Theileria* infection in ticks and large ruminants. These results can help to develop more practical methods for preventing and controlling bovine tropical theileriosis, which will ultimately improve the welfare of animals and the standard of living for farmers.

## Figures and Tables

**Table 1:** Frequency distribution of hard ticks (Acari: Ixodidae) in large ruminants of Toba Tek Singh district, Punjab, Pakistan.

Levels	Positive	Prevalence (%)	Odds Ratio	*p*-Value	95% Confidence Interval
*Hyalomma anatolicum*	136	35.4	1.39	0.000	41.56–45.54
*Rhipicephalus (Boophilus) microplus*	116	30.2	1.18	0.001	27.41–31.19
*Rhipicephalus sanguineus*	96	25	-	-	22.20–25.66

*n* = 384 (total number of animals examined).

**Table 2:** Frequency distribution of *Theileria* (Order: Family) infection in the salivary glands of *Hyalomma anatolicum* (Acari: Ixodidae) ticks collected from Toba Tek Singh district, Punjab, Pakistan.

Variables	Levels	Examined	Positive	Odds Ratio	*p*-Value	95% Confidence Interval
Host	Non-host	116	42	-	-	21.61–51.17
	Buffalo ticks	66	24	2.47	0.029	20.30–52.51
	Cattle ticks	70	60	1.37	0.000	70.03–93.46
Sex and Infestation Status	Male ticks (infesting)	62	36	4.49	0.007	41.24–73.22
	Female ticks (infesting)	74	48	5.14	0.003	47.50–77.76
	Male ticks (questing)	62	8	-	-	03.13–27.14
	Female ticks (questing)	54	34	4.76	0.006	42.74–78.31
Ecological Variable	Non-riverine	126	40	-	-	20.06–42.85
	Riverine	126	86	2.04	0.000	55.13–77.72

**Table 3:** Association of various risk factors with *Theileria* infection in blood collected from large ruminants of Toba Tek Singh, Punjab, Pakistan.

Variables	Levels	Animal Examined	Positive	Prevalence (%)	Odds Ratio	*p*-Value	95% Confidence Interval
Species	Cattle	192	83	43.2	1.12	0.001	41.58–45.26
	Buffalo	192	55	28.6			25.79–31.25
Cattle breed	Friesian	38	22	57.9	1.42	0.002	54.45–60.38
	Jersey	69	31	44.9	2.34	0.043	42.16–47.36
	Sahiwal	85	30	35.3	-	-	33.16–37.51
Buffalo breeds	Nili Ravi	114	20	17.5	2.18	0.000	15.38–19.04
	Kundi	78	35	44.9			41.65–46.64
Cattle sex	Male	46	13	28.3	2.19	0.000	25.78–30.51
	Female	146	70	47.9			45.62–50.25
Buffalo sex	Male	37	6	16.2	1.86	0.000	14.67–20.64
	Female	155	49	31.6			29.38–33.34
Cattle age	Young	70	39	55.7	1.49	0.000	53.44–58.41
	Adult	122	44	36.1			33.08–38.05
Buffalo age	Young	62	25	40.3	1.37	0.000	38.37–42.34
	Adult	130	30	23.1			21.72–25.79
Cattle	Riverine	92	48	52.2	1.98	0.000	49.09–54.36
	Non-riverine	100	35	35			32.37–37.43
Buffalo	Riverine	105	38	36.2	2.12	0.000	34.94–38.91
	Non-riverine	87	17	19.5			17.74–21.57

## Data Availability

All the data can be found in the main text.

## References

[1] Asmaa N.M., El Bably M.A., Shokier K.A. (2014). Studies on prevalence, risk indicators and control options for tick infestation in ruminants. Beni-Suef Univ. J. Basic. Appl. Sci..

[2] Hasan U.M., Abubakar M., Muhammad G., Khan M.N., Hussain M. (2012). Prevalence of tick infestation (*Rhipicephalus sanguineus* and *Hyalomma anatolicum anatolicum*) in dogs in Punjab, Pakistan. Vet. Ital..

[3] Yamchi J.A., Tavassoli M. (2016). Survey on infection rate, vectors and molecular identification of *Theileria annulata* in cattle from North West, Iran. J. Parasit. Dis..

[4] Rehman A., Nijhof A.M., Sauter-Louis C., Schauer B., Staubach C., Conraths F.J. (2017). Distribution of ticks infesting ruminants and risk factors associated with high tick prevalence in livestock farms in the semi-arid and arid agro-ecological zones of Pakistan. Parasites Vectors.

[5] Perveen F. (2011). Distribution and identification of Ixodid tick species on livestock in Northern Pakistan. J. Agric. Sci. Technol..

[6] Karim S., Budachetri K., Mukherjee N., Williams J., Kausar A., Hassan M.J., Adamson S., Dowd S.E., Apanskevich D., Arijo A. (2017). A study of ticks and tick-borne livestock pathogens in Pakistan. PLoS Negl. Trop. Dis..

[7] Farooqi S.H., Ijaz M., Saleem M.H., Rashid I., Oneeb M., Khan A., Aqib A.I., Mahmood S. (2017). Distribution of ixodid tick species and associated risk factors in temporal zones of Khyber Pakhtunkhawa Province, Pakistan. Pak. J. Zool..

[8] Amiri M.S., Yaghfoori S., Razmi G. (2021). Molecular detection of *Theileria annulata* among dairy cattle and vector ticks in the Herat Area, Afghanistan. Arch. Razi Inst..

[9] Zeb J., Song B., Aziz M.U., Hussain S., Zarin R., Sparagano O. (2022). Diversity and distribution of *Theileria* species and their vectors in ruminants from India, Pakistan and Bangladesh. Diversity.

[10] Jabbar A., Abbas T., Sandhu Z.U.D., Saddiqi H.A., Qamar M.F., Gasser R.B. (2015). Tick-borne diseases of bovines in Pakistan: Major scope for future research and improved control. Parasit. Vectors.

[11] Fry L.M., Schneider D.A., Frevert C.W., Nelson D.D., Morrison W.I., Knowles D.P. (2016). East coast fever caused by *Theileria parva* is characterized by macrophage activation associated with vasculitis and respiratory failure. PLoS ONE.

[12] Islam M.K., Jabbar A., Campbell B.E., Cantacessi C., Gasser R.B. (2011). Bovine theileriosis—An emerging problem in south-eastern Australia?. Infect. Genet. Evol..

[13] Brown C.G.D. (1997). Dynamics and impact of tick-borne diseases of cattle. Trop. Anim. Health Prod..

[14] De La Fuente J., Estrada-Pena A., Venzal J.M., Kocan K.M., Sonenshine D.E. (2008). Overview: Ticks as vectors of pathogens that cause disease in humans and animals. Front. Biosci..

[15] Walker A.R., Bouattour A., Camicas J.L., Estrada-Pena A., Horak I.G., Latif A.A., Pegram R.G., Preston P.M. (2003). Ticks of Domestic Animals in Africa: A Guide to Identification of Species.

[16] Purnell R.E., Joyner L.P. (1968). The development of *Theileria parva* in the salivary glands of the tick *Rhipicephalus appendiculatus*. Parasitology.

[17] Allsopp B.A., Baylis H.A., Allsopp M.T., Cavalier-Smith T., Bishop R.P., Carrington D.M., Sohanpal B., Spooner P. (1993). Discrimination between six species of *Theileria* using oligonucleotide probes which detect small subunit ribosomal RNA sequences. Parasitol..

[18] d’Oliveira C.M., van der Weide M., Habela M.A., Jacquiet P., Jongejan F. (1995). Detection of *Theileria annulata* in blood samples of carrier cattle by PCR. J. Clin. Micro.

[19] Zeb J., Shams S., Din I.U., Ayaz S., Khan A., Nasreen N., Khan H., Khan M.A., Senbill H. (2020). Molecular epidemiology and associated risk factors of *Anaplasma marginale* and *Theileria annulata* in cattle from North-western Pakistan. Vet. Parasitol..

[20] Rehman A., Conraths F.J., Sauter-Louis C., Krücken J., Nijhof A.M. (2019). Epidemiology of tick-borne pathogens in the semi-arid and the arid agro-ecological zones of Punjab province, Pakistan. Transbound. Emerg. Dis..

[21] Batool A., Sajid M.S., Rizwan H.M., Iqbal A., Rashid I., Jan I., Bano F., Ahmad F., Ahmad W., Khan M.N. (2022). Association of various risk factors with the distribution of gastrointestinal, haemo and ectoparasites in small ruminants. J. Anim. Health Prod..

[22] Morrison W.I., Hemmink J.D., Toye P.G. (2020). *Theileria parva*: A parasite of African buffalo, which has adapted to infect and undergo transmission in cattle. Int. J. Parasitol..

[23] Liu J., Guan G., Yin H. (2021). *Theileria annulata*. Trends Parasitol..

[24] Almazán C., Scimeca R.C., Reichard M.V., Mosqueda J. (2022). Babesiosis and theileriosis in North America. Pathogens.

[25] Khan M.N., Hayat C.S., Iqbal Z., Hayat B., Naseem A. (1993). Prevalence of ticks on livestock in Faisalabad Pakistan. Pak. Vet. J..

[26] Sajid M.S., Iqbal Z., Khan M.N., Muhammad G., Khan M.K. (2009). Prevalence and associated risk factors for bovine tick infestation in two districts of lower Punjab, Pakistan. Prevent Vet. Med..

[27] Siddiqi M.N., Jan A.H. (1986). Ixodid ticks Ixodidae of N.W.F.P. (North-West Frontier Province) Pakistan. Pak. Vet. J..

[28] Hussain S.I., Kumar G.A. (1986). The incidence of ticks (Ixodidea: Ixodidae) infesting sheep and goats in Sind province, Pakistan. Pak. J. Zool..

[29] Iqbal A., Sajid M.S., Khan M.N., Khan M.K. (2013). Frequency distribution of hard ticks (Acari: Ixodidae) infesting bubaline population of district Toba Tek Singh, Punjab, Pakistan. Parasitol. Res..

[30] Kocan A.A., Kocan K.M., Karr C.H., Hair J.A. (1988). Electron microscopic observations of *Theileria cervi* in salivary glands of male *Amblyomma americanum*. Proc. Helminthol. Soc. Wash..

[31] Woods K., Perry C., Brühlmann F., Olias P. (2021). Theileria’s strategies and effector mechanisms for host cell transformation: From invasion to immortalization. Front. Cell Dev. Biol..

[32] Kolte S.W., Larcombe S.D., Jadhao S.G., Magar S.P., Warthi G., Kurkure N.V., Glass E.J., Shiels R. (2017). PCR diagnosis of tick-borne pathogens in Maharashtra state, India indicates fitness cost associated with carrier infections is greater for crossbreed than native cattle breeds. PLoS ONE.

[33] Kumar B., Manjunathachar H.V., Ghosh S. (2020). A review on *Hyalomma* species infestations on human and animals and progress on management strategies. Heliyon.

[34] Haque M., Jyoti Singh N.K., Rath S.S. (2010). Prevalence of *Theileria annulata* infection in *Hyalomma anatolicum anatolicum* in Punjab state, India. J. Parasitol. Dis..

[35] Bishop R.B., Odongo D.A., Mann D.J., Pearson T.W., Sugimoto C., Haines L.R., Glass E.J., Jensen K., Seitzer U., Ahmed J.S., Nen V., Kole C. (2009). Theileria. Genome Mapping and Genomics in Animal-Associated Microbes.

[36] Farooqi S., Ijaz M., Saleem M., Rashid M., Ahmad S., Islam S., Aqib A.I., Khan A., Hussain K., Khan N.U. (2017). Prevalence and molecular diagnosis of *Theileria annulata* in bovine from three distinct zones of Khyber Pakhtunkhwa province, Pakistan. J. Anim. Plant Sci..

[37] Qayyum M., Farooq U., Samad H.A., Chauhdry H.R. (2010). Prevalence, clinicotherapeutic and prophylactic studies on theileriosis in district Sahiwal (Pakistan). J. Anim. Plant Sci..

[38] Ullah R., Shams S., Khan M.A., Ayaz S., Akbar N.U., Din Q.U., Khan A., Leon R., Zeb J. (2021). Epidemiology and molecular characterization of *Theileria annulata* in cattle from central Khyber Pakhtunkhwa, Pakistan. PLoS ONE.

[39] Kabir M.H., Mondal M.M., Eliyas M., Mannan M.A., Hashem M.A., Debnath N.C., Miazi O.F., Mohiuddin C., Kashem M.A., Islam M.R. (2011). An epidemiological survey on investigation of tick infestation in cattle at Chittagong District, Bangladesh. Advan J. Microbiol. Res..

[40] Ilhan T., Williamson S., Kirvar E., Shiels B., Brown C. (1998). *Theileria annulata*: Carrier state and immunity. Ann. N. Y. Acad. Sci..

[41] Gharbi M., Darghouth M.A. (2014). A review of *Hyalomma scupense* (Acari, Ixodidae) in the Maghreb region: From biology to control. Parasite.

[42] Valente D., Dutra A.P., Carolino N., Gomes J., Coelho A.C., Espadinha P., Pais J., Carolino I. (2023). Prevalence and risk factors associated with *Theileria annulata* infection in two bovine Portuguese autochthonous breeds. Pathogens.

[43] Kamani J., Sannusi A., Egwu O.K., Dogo G.I., Tanko T.J., Kemza S., Tafarki A., Gbise D. (2010). Prevalence and significance of haemoparasitic infections of cattle in North- Central Nigeria. Vet. World.

[44] Parveen A., Alkhaibari A.M., Asif M., Almohammed H.I., Naqvi Z., Khan A., Aktas M., Ozubek S., Farooq M., Iqbal F. (2021). Molecular epidemiology of *Theileria annulata* in cattle from two districts in Punjab (Pakistan). Animals.

[45] Magzoub A., El Ghali A., Hussien M.O., Juma Y., Mohammed S.B. (2021). Prevalence of ticks (Acari: Ixodidae) and *Theileria lestoquardi* in sheep at El Huda and El Nuhud animal production research stations, Sudan. J. Parasit. Dis..

[46] Salih D.A., El Hussein A.M., Hayat M., Taha K.M. (2003). Survey of *Theileria lestoquardi* antibodies among Sudanese sheep. Vet. Parasitol..

[47] Iweriebor B.C., Afolabi K.O., Nqoro A., Obi L.C. (2022). Emergence of *Theileria* species in ticks from free-ranging domestic animals in Raymond Mhlaba local municipality, South Africa. Heliyon.

